# Isomerization of an Antimicrobial Peptide Broadens Antimicrobial Spectrum to Gram-Positive Bacterial Pathogens

**DOI:** 10.1371/journal.pone.0046259

**Published:** 2012-10-02

**Authors:** Chiara Falciani, Luisa Lozzi, Simona Pollini, Vincenzo Luca, Veronica Carnicelli, Jlenia Brunetti, Barbara Lelli, Stefano Bindi, Silvia Scali, Antonio Di Giulio, Gian Maria Rossolini, Maria Luisa Mangoni, Luisa Bracci, Alessandro Pini

**Affiliations:** 1 Dipartimento di Biotecnologie Mediche, Università degli Studi di Siena, Siena, Italy; 2 Dipartimento di Scienze Biochimiche A. Fanelli, Università di Roma, La Sapienza, Roma, Italy; 3 Dipartimento di Scienze e Tecnologie Biomediche, Università di L’Aquila, L’Aquila, Italy; 4 SetLance srl, Siena, Italy; 5 Azienda Ospedaliera Universitaria Senese, Policlinico Le Scotte, Siena, Italy; Charité-University Medicine Berlin, Germany

## Abstract

The branched M33 antimicrobial peptide was previously shown to be very active against Gram-negative bacterial pathogens, including multidrug-resistant strains. In an attempt to produce back-up molecules, we synthesized an M33 peptide isomer consisting of D-aminoacids (M33-D). This isomeric version showed 4 to 16-fold higher activity against Gram-positive pathogens, including *Staphylococcus aureus* and *Staphylococcus epidermidis,* than the original peptide, while retaining strong activity against Gram-negative bacteria. The antimicrobial activity of both peptides was influenced by their differential sensitivity to bacterial proteases. The better activity shown by M33-D against *S. aureus* compared to M33-L was confirmed in biofilm eradication experiments where M33-L showed 12% activity with respect to M33-D, and *in vivo* models where Balb-c mice infected with *S. aureus* showed 100% and 0% survival when treated with M33-D and M33-L, respectively. M33-D appears to be an interesting candidate for the development of novel broad-spectrum antimicrobials active against bacterial pathogens of clinical importance.

## Introduction

Antimicrobial resistance (AMR) is not a recent phenomenon, but it is a critical health issue today. Over several decades, to varying degrees, bacteria causing common infections have developed resistance to each new antibiotic, and AMR has evolved to become a worldwide health threat. With a dearth of new antibiotics coming to market, the need for action to avert a developing global crisis in health care is increasingly urgent [1]. Antimicrobial peptides (AMPs) are seen with great interest for the development of new agents against bacterial infections, because most of them show strong bactericidal activity against multidrug-resistant (MDR) bacterial pathogens, and may also contribute to innate immunity by modulating dendritic cell differentiation and maturation, angiogenesis and chemokine production [2]. These features are particularly attractive and many natural host defense peptides (HDPs) or artificial AMPs are currently under experimentation for drug development [3]. Unfortunately, certain drawbacks have limited the development of AMPs as drugs for bacterial infections: i) toxicity to eukaryotic cells, that may lead to nephrotoxicity, neurotoxicity and neuromuscular blockade [4], [5]; ii) selection of resistant strains that may be cross-resistant to human-neutrophil-defensin-1, a key component of the innate immune response to infection [6]; iii) the fact that natural AMPs are generally very short peptides easily attacked by circulating proteolytic enzymes, making their half-life too short to be active against bacteria *in vivo*. Researchers and industry have been seeking new AMPs of natural and non-natural origin, with low toxicity and the longer half-life necessary for drug development.

A few years ago, we observed that short peptides synthesized in oligodendrimeric form [7] showed high resistance to proteolytic degradation, making them suitable for use *in vivo* [8]–[10]. The synthetic peptide M33 was obtained by random selection from a home-made phage-display peptide library panned against *E. coli* cells and a successive optimization phase for biological activity, synthesis and purification procedures [11]–[14]. The M33 sequence (KKIRVRLSA) is amphipathic and cationic, which is typical for AMPs, but did not show any sequence homology with known AMPs of natural or non-natural origin. M33 was synthesized in tetra-branched form, proving resistant to proteolytic degradation and very active *in vitro* against clinical isolates of several Gram-negative pathogens, including MDR strains of *Pseudomonas aeruginosa*, *Acinetobacter baumannii*, *Klebsiella pneumoniae* and *Escherichia coli*, while being less active against the Gram-positive pathogen *Staphylococcus aureus*. The peptide also protected mice lethally infected with multi-resistant clinical isolates of *P. aeruginosa* and is currently under preclinical characterization for the development of a new drug for bloodstream and lower respiratory tract infections.

In previous reports [11]–[14] the peptide was always synthesized and used with L aminoacids (M33-L). Recently, we used the same sequence synthesized in the tetra-branched form using D aminoacids (M33-D). Here we report that compared to M33-L, M33-D has stronger activity against *S. aureus* and coagulase-negative staphylococci, including methicillin-resistant strains, with MIC values comparable to those of many antimicrobial agents used in clinical practice. We also report a study of the mechanism of action of M33-D compared to M33-L. Since M33-D retains strong activity against Gram-negative pathogens, it appears to be an interesting candidate for the development of novel broad-spectrum AMPs.

## Results and Discussion

### MIC Determination

MICs of M33-L and M33-D were determined against strains of different bacterial species, including major Gram-negative and Gram-positive pathogens (Table 1). Compared to M33-L, M33-D exhibited the same activity against *P. aeruginosa* and the same or a slightly lower (2–4 fold) activity against Enterobacteriaceae. On the other hand, M33-D showed higher antimicrobial activity than M33-L against the Gram-positive bacteria *S. aureus* and *S. epidermidis*, including methicillin-resistant and vancomycin-intermediate strains, with MICs 4 to 16-fold lower than those of M33-L. As previously observed with M33-L [13], M33-D exhibited antimicrobial activity (MIC values) against antibiotic-susceptible reference bacterial strains and MDR strains of clinical origin expressing several different mechanisms of antibiotic resistance.

**Table 1:** MICs of M33-L and M33-D for different bacteria species and strains.

Species and strains	Relevant features^a^	M33-L (µM)	M33-D (µM)
*P.aeruginosa ATCC 27853*	Reference strain, wild type	1.5	1.5
*P.aeruginosa AV 65*	FQ^r^ AG^r^ ESC^r^ NEM^r^ (MBL/IMP-13)	3	3
*K.pneumoniae ATCC 13833*	Reference strain, wild type	1.5	3
*K.pneumoniae 7086042*	FQ^r^ AG^r^ ESC^r^ NEM^r^ (MBL/VIM-1)	3	6
*E.coli ATCC 25922*	Reference strain, wild type	3	3
*E.coli W03BG0025*	FQ^r^ AG^r^ ESC^r^ (ESBL/CTX-M-15)	0.7	3
*S.aureus ATCC 29213*	Reference strain, wild type	6	1.5
*S.aureus USA 300*	MR	6	1.5
*S.aureus 3851*	MR VAN^i^	12	0.7
*S.epidermidis ATCC 14990*	Reference strain, wild type	1.5	0.4
*S.epidermidis 6154*	MR	3	0.7

a M33 antimicrobial activity was evaluated on reference strains and clinical isolates (mostly with an MDR phenotype). Relevant resistance phenotypes and resistance determinants are indicated. Resistance phenotypes: FQ^r^, resistant to fluoroquinolones; AG^r^, resistant to aminoglycosides (gentamycin, amikacin, and/or tobramycin); ESC^r^, resistant to expanded-spectrum cephalosporins; NEM^r^, resistant to carbapenems (imipenem and/or meropenem); MR^r^, methicillin-resistant; VAN^i^, vancomycin-intermediate. Resistance determinants: ESBL, extended spectrum β-lactamase; MBL, metallo-β-lactamase.

### Binding of M33-L and M33-D to Lipopolysaccharide (LPS) and Lipoteichoic Acid (LTA)

**Figure 1:**
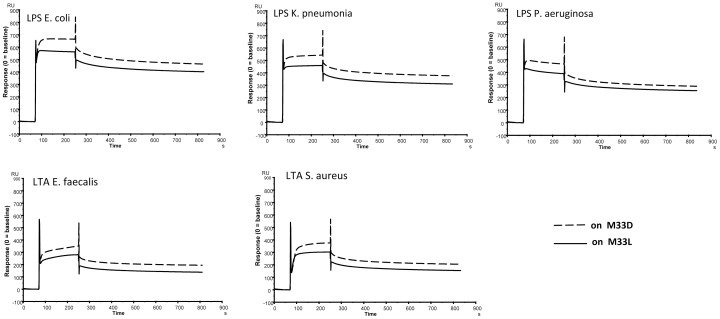
Binding of LTA and LPS on M33-L or M33-D measured by surface plasmon resonance. LPS from *P. aeruginosa*, *K. pneumoniae*, *E. coli* and LTA from S. *faecalis* and *S. aureus*, diluted to 10 µg/ml were injected over M33-L and M33-D immobilized peptides.

In a previous report [13] we hypothesized that LPS was the first bacterial structure to interact with M33-L. In order to evaluate possible differential binding of M33-L and M33-D to Gram-negative LPS and to Gram-positive LTA, we therefore analyzed the interactions of both peptides with LPS and LTA by surface plasmon resonance. LTA from *S. aureus* and *Streptococcus faecalis*, and LPS from *E. coli*, *P. aeruginosa* and *K. pneumoniae* were injected at a concentration of 10 µg/ml over immobilized M33-L or M33-D peptides. No significant difference in binding or kinetic rates that could explain such dissimilar antimicrobial activity of the two peptides was observed (Fig. 1).

### Interaction of M33 with Liposomes Mimicking Bacterial Cells

**Figure 2:**
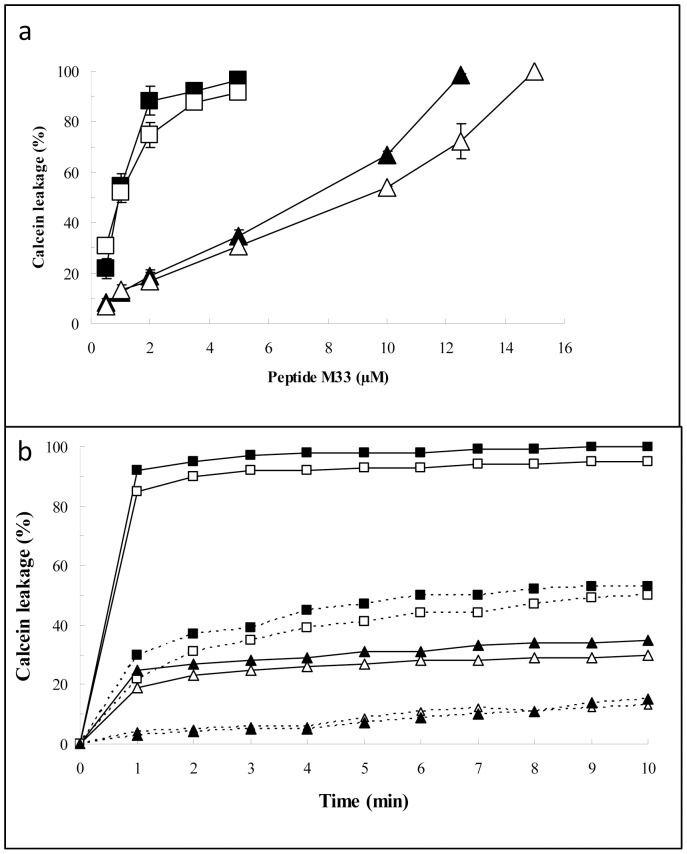
Release of calcein from bacterial-surface-mimicking liposomes. a , dose-response of M33-induced calcein release**.** The vesicles were incubated with different concentration of M33 peptide for 10 min at 20°C (for details see Methods section). CL/PG liposomes (triangles); PE/PG liposomes (squares); M33-D: full symbols; M33-L: empty symbols. Values are means ± SE of three independent experiments. **b**, time course of calcein release from: CL/PG liposomes (triangles) and from PE/PG liposomes (squares); M33-D: full symbols; M33-L: empty symbols. continuous line 5 µM, dotted line 1 µM.

To investigate interaction of peptides M33-D and M33-L with the bacterial membrane, including possible perturbation, we used vesicles with two lipid compositions to mimic the membrane of *S. aureus* (CL/PG, 4∶6 mol/mol) and *E. coli* (PE/PG, 7∶3 mol/mol) [15]. Both liposome preparations were treated with increasing peptide concentrations from 0,5 to 15 µM and the membrane permeability was revealed by measuring the fluorescence increase due to the calcein leakage from the vesicles. The dose-response curves obtained from CL/PG or PE/PG liposomes are reported in Fig. 2a. The peptide-induced effect was dose-dependent in both vesicle lipid compositions. However, effectiveness on the two lipid compositions was significantly different, since maximum calcein release from CL/PG liposomes was obtained at peptide concentrations greater than 10 µM, whereas in PE/PG liposomes total leakage occurred at peptide concentration of 5 µM. No significant differences in the effects induced by M33-D and M33-L were evident, although the D peptide seemed slightly more efficient towards CL/PG liposomes at doses above 8 µM. Fig. 2b shows the time-course of probe release when the vesicles were treated with M33-D or M33-L at 1 or 5 µM final concentrations. In all cases, the peptide-induced increase in fluorescence showed a typical biphasic kinetic profile, in which a fast phase due to the initial membrane-peptide interaction was followed by a slow steady-state. The greater perturbing effect of both forms of M33 on PE/PG vesicles, compared to vesicles containing cardiolipin, was evident.

These tests, along with the Biacore analysis described above, revealed that M33-D and M33-L have substantially similar behavior in terms of binding to LPS and LTA and of perturbation of membranes of different phospholipid composition. We deduced that the mechanism used by M33-L and M33-D for interacting with bacterial surfaces and disruption of bacterial membranes was basically the same.

### Stability to Bacterial Proteases

**Figure 3:**
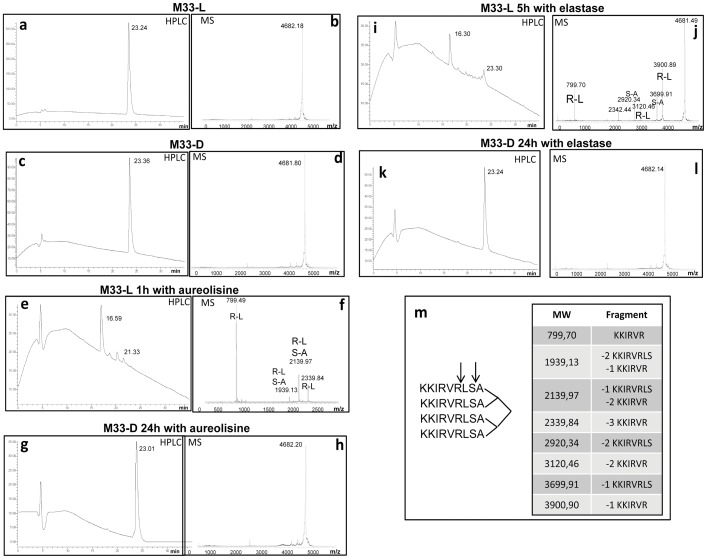
Proteolytic activity of aureolysin and elastase on peptides M33-L and M33-D. **a** and **b**, HPLC and MS profiles, respectively, of M33-L before incubation with enzymes. **c** and **d**, HPLC and MS profiles, respectively, of M33-D before incubation with enzymes. In HPLC the retention time of M33-L and M33-D was 23 minutes. The calculated MW of M33 was 4682. **e** and **f**, HPLC and MS, respectively, of M33-L incubated for 1 hour with aureolysin. **f** shows the peaks indicating the proteolytic site (RL or SA). **g** and **h**, HPLC and MS, respectively, of M33-D incubated for 24 hours with aureolysin. **i** and **j**, HPLC and MS, respectively, of M33-L incubated for 5 hours with elastase. **j** shows the peaks indicating the proteolytic site (RL or SA). **k** and **l**, HPLC and MS, respectively, of M33-D incubated for 24 hours with elastase. **m**, proteolytic sites of the two enzymes on the tetrabranched M33 are indicated by arrows. The table assigns MS peaks to the cleavage fragments.

Peptide stability to bacterial proteases was analyzed with purified aureolysin and elastase enzymes derived from *S. aureus* and *P. aeruginosa*, respectively. These proteins play a key role in bacterial virulence by breaking down natural HDPs produced by the infected individuals [16]–[18]. *S. aureus* aureolysin and *P. aeruginosa* elastase are members of the family of M4 metallopeptidases (thermolysin family) [19]–[21] and have similar specificity, hydrolyzing peptide bonds preferentially on the amino-terminal side of hydrophobic residues. To determine whether these proteases affect the performance of M33 peptides, M33-L and M33-D were incubated with aureolysin and elastase, respectively, and after appropriate time intervals the crude solutions were analyzed by HPLC and mass spectroscopy. Unlike M33 incubated without enzymes (Figs. 3a, 3b, 3c and 3d), M33-L was degraded within 1h by staphylococcal aureolysin (Fig. 3e), through hydrolysis at R6-L7 and S8-A9 peptide bonds (Fig. 3f). Conversely, M33-D was completely stable to proteolysis by this metalloprotease, remaining unaltered after 24 h of incubation (Figs. 3g and 3h). Incubation of M33-L with *P. aeruginosa* elastase showed moderate peptide stability after 5 h (a peak corresponding to a retention time of 23 min is still present in Fig. 3i), and again the cleavage sites were R6-L7 and S8-A9 peptide bonds (Fig. 3j). In contrast, the M33-D peptide resisted degradation by elastase for 24 h (Figs. 3k and 3l). The cleavage sites of both peptides are illustrated in Fig. 3m and the MS peaks are assigned to the fragments.

Altogether, these results suggest that the increased stability of M33-D to staphylococcal aureolysin could be at least partly responsible for the increased activity exhibited by this isomer against *S. aureus.* The same phenomenon could also explain the increased activity of M33-D against *S. epidermidis* (Table 1), which produces an ortholog of aureolysin (the metallo-protease SepA, with 71% aminoacid identity [22]–[23]).

### Assessment of Anti-biofilm Activity

In biofilms, bacteria grow as multicellular aggregates within an extracellular matrix that protects the cells from host defences. Biofilms are also more resistant to antimicrobial agents due to the physiological state of bacterial cells and, in some cases, reduced antibiotic penetration [24]. Bacterial biofilms form in natural, medical and industrial settings, and play a major role in several human infections, including infections of prosthetic devices and intravascular catheters, bone and joint infections, chronic rhinosinusitis and otitis media [25], [26]. The search for new antimicrobials that eradicate microbial biofilms has therefore become extremely pressing.

M33-L and M33-D were tested for their anti-biofilm activity against the Gram-negative strains *E. coli* ATCC 25922 and *P. aeruginosa* ATCC 27853, as well as the Gram-positive strain *S. aureus* ATCC 25923. As reported in Table 2, the minimum biofilm eradication concentrations (MBECs) of the two peptides observed with Gram-negatives were on the whole similar. On the other hand, M33-D exhibited higher anti-biofilm activity against *S. aureus* than M33-L (MBEC, 1.5 µM vs. 12 µM), which is consistent with the difference in MIC of the two isomers for this strain (Table 1). The minimum bactericidal concentration on biofilm (MBCb), i.e. the concentration that kills 99.9% of biofilm cells, was also investigated. The two isomers showed an MBCb of 6 µM against the Gram-negatives *E. coli* and *P. aeruginosa* (Table 2), whereas the MBCbs of M33-L and M33-D for the Gram-positive *S. aureus* matched the respective MBECs, being 12 and 1.5 µM, respectively.

**Table 2:** Anti-biofilm activity of M33-L and M33-D towards different bacterial species.

Bacterial species	Minimum biofilm eradication concentration (MBEC, µM)^a^		Minimum bactericidal concentration on biofilm (MBCb, µM)^b^	
	M33-L	M33-D	M33-L	M33-D
***Gram-negatives***				
*E. coli ATCC 25922*	3	3	6	6
*P. aeruginosa ATCC 27853*	1.5	3	6	6
***Gram-positive***				
*S. aureus ATCC 25923*	12	1.5	12	1.5

a MBEC is the minimum peptide concentration preventing regrowth of bacteria from the treated biofilm within 4 hours.

b MBCb is the minimum peptide concentration required to reduce the number of viable biofilm cells by ≥3 log_10_ (99.9% killing) after 2 h.

### *In vivo* Anti-MRSA Activity of M33-D *vs.* M33-L

Given the good *in vitro* activity shown by M33-D against methicillin-resistant *S. aureus* (MRSA), we compared the *in vivo* activity of this peptide and the original M33-L in an animal model of infection caused by the highly virulent MRSA strain USA 300, a lineage that has become a dominant cause of community-associated MRSA infections in North America [27], [28].

**Figure 4:**
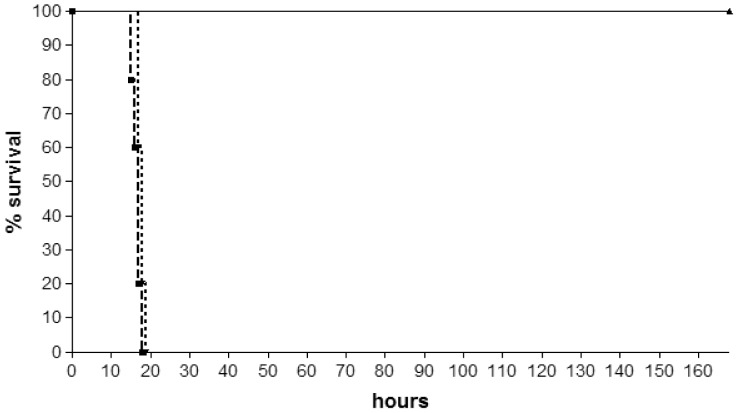
*In vivo* antibacterial activity of tetrabranched M33-L and M33-D peptides. Balb-c mice (20 g) were injected i.p. with a lethal amount of *S. aureus* USA300 cells. Dashed line (Ctr), injection with bacteria and no peptides; dotted line, injection with bacteria and a single injection of M33-L peptide (25 mg/kg) 30 min later; continuous line, injection with bacteria and a single injection of M33-D peptide (25 mg/kg) 30 min later.

The smallest number of bacteria causing 100% lethal infection (LD100) after intra-peritoneal (i.p.) injection was 1×10^6^ in the presence of 7% mucin. An LD100 killed mice within 20 hours. Mice were infected with the LD100 of bacteria and treated i.p. with the peptides 30 minutes later. 100% survival after 7 days was obtained with mice treated with M33-D, while mice treated with M33-L showed a mortality overlapping that of controls (Fig. 4), confirming the potent anti-MRSA activity of M33-D.

### Conclusions

The M33 peptide, previously reported as active against a broad spectrum of Gram-negative bacteria [13], is also strongly active against staphylococci when synthesized with D-aminoacids. We hypothesized that the increased stability of M33-D to staphylococcal proteases could at least partly explain this different activity. It was known that branched peptides, like those used in this study, are particularly resistant to circulating proteases produced by higher animals [8]–[10], [29]–[31]. It was also known that peptides with D-aminoacids show increased stability to circulating proteases [32]. Stability of D-peptides to bacterial proteases has also been reported [33], [34]. In our case the concomitant improvement of stability of M33-D to infected individual proteases and to infectious agent proteases dramatically increased the overall performance of the peptide. This is particularly evident in experiments *in vivo* where M33-D neutralized signs of sepsis due to *S. aureus* USA300, while M33-L, stable to mouse [13] but not to bacterial proteases, was not active at all.

M33-D was highly stable to the proteases aureolysin, from *S. aureus*, and elastase, from *P. aeruginosa*. M33-L was not at all stable to aureolysin and poorly stable to elastase, as confirmed by its activity *in vivo* against *P. aeruginosa* [13]. For M33-D we propose the following mechanism of action. M33-D binds LTA and persists on the bacterial surface for some time by virtue of its resistance to bacterial proteases, causing membrane perturbation that kills the bacteria.

Concluding, we identified a new form of the peptide M33, which is strongly active against *S. aureus* and retains its antimicrobial activity irrespective of strain-resistance phenotypes and mechanisms. MRSA and *S. aureus* strains with altered susceptibility to glycopeptides pose a serious clinical threat and major therapeutic challenge. In this context, development of a new broad-spectrum therapeutic agent with no cross-resistance to available drugs would be a major achievement.

## Materials and Methods

### Peptide Synthesis

Solid-phase synthesis was carried out by standard Fmoc chemistry on Fmoc4-Lys2-Lys-β-Ala Wang resin with a Syro multiple peptide synthesizer (MultiSynTech, Witten, Germany). Side chain protecting groups were 2,2,4,6,7-pentamethyldihydrobenzofuran-5-sulfonyl for R, t-butoxycarbonyl for K and t-butyl for S. M33-L was synthesized using Fmoc-L-aminoacids, and M33-D with Fmoc-D-aminoacids with the exception of the three lysins of the branched core which were Fmoc-L-Lys(Fmoc)-OH (M33-D is consequently a diastereomer). The final products were cleaved from the solid support, deprotected by treatment with TFA containing triisopropylsilane and water (95/2.5/2.5), and precipitated with diethyl ether. Crude peptides were purified by reversed-phase chromatography on a Phenomenex Jupiter C18 column (300 Å, 10 µm, 250×10 mm) in linear gradient form for 30 min, using 0.1% TFA/water as eluent A and methanol as eluent B. Purified peptides were obtained as trifluoroacetate salts (TFacetate). The exchange from TFacetate to acetate form was carried out using a quaternary ammonium resin in acetate form (AG1-X8, 100–200 mesh, 1.2 meq/ml capacity, Bio-Rad). The resin-to-peptide ratio was 2000∶1, resin and peptide were stirred for 1 h, the resin was filtered off, washed extensively and the peptide recovered and freeze-dried. Final peptide purity and identity were confirmed by reversed phase chromatography on a Phenomenex Jupiter C18 analytical column (300 Å, 5 µm, 250×4.6 mm) and by mass spectrometry with a Bruker Daltonics ultraflex MALDI TOF/TOF.

### MIC Testing

MICs were determined using a standard microdilution assay as recommended by the Clinical and Laboratory Standards Institute. Assays were performed in triplicate using cation-supplemented Mueller-Hinton (MH) broth (Becton Dickinson, Franklin Lakes, NJ, USA) and a bacterial inoculum of 5x10^4^ CFU/well, in a final volume of 100 µl. The tested concentrations ranged from 0.1 µM to 24 µM for both peptides. Results were recorded after 18–20 h of incubation at 37°C.

### Surface Plasmon Resonance

Biotinylated peptides were immobilized on SA coated flow cells. M33-L and M33-D peptides, diluted to 10 µg/ml in HBS-EP+ buffer (10 mM Hepes, 150 mM NaCl, 3.4 mM EDTA, 0.05% polysorbate 20 pH 7.4), were injected for 90 sec at a flow rate of 10 µl/min, obtaining 550 RU and 580 RU for M33-L and M33-D respectively.

LTA and LPS molecules from different species (LPS from *E. coli*, *K. pneumonia*, *P. aeruginosa* and LTA from *S. aureus* and *S. faecalis*, were obtained from Sigma-Aldrich: L-3012, L-4268, L9143, L2515 and L4015, respectively) were diluted in HBS-EP+ buffer at the concentration of 10 µg/ml and injected for 180 sec with a flow rate of 30 µl/min over immobilized peptides. An empty flow cell was used as reference. Regeneration was achieved with a short pulse of SDS 0.05%.

### Preparation of Calcein-liposomes and Leakage Measurement

L-α-phosphatidylethanolamine (PE), L-α-phosphatidyl-DL-glycerol (PG), cardiolipin (CL), calcein, ammonium thiocyanate and iron (III) chloride hexahydrate and all other chemical (reagent grade) were obtained from Sigma.

Calcein-loaded liposomes of two different composition (PE/PG, 7∶3 mol/mol and CL/PG, 4∶6 mol/mol) were prepared as follows. The lipids were dissolved in chloroform (1 ml) and sonicated together with 60 mM calcein solution (1 ml in phosphate buffer, pH 7.0); the liposomes were obtained by the reverse phase evaporation method [35]. The calcein excess was removed by gel filtration (Sephadex G-50) followed by centrifuging at 22000 *g* for 30 min. For vesicle size homogeneity, the pellet was passed several times through 200 µm polycarbonate membranes in a Mini-extruder apparatus (Avanti Polar Lipids Inc., Alabaster AL) [36]. Lipid concentration of vesicles was measured by the method of Stewart [37] and the final concentration used for all measurements was 50 µM. Calcein fluorescence in the vesicles is self-quenched and leakage was measured by relief of quenching; the measurements were carried out at 517 nm, exciting at 490 nm, with a Perkin-Elmer LS 50B spectrofluorimeter. The maximum value of leakage was obtained by addition of 10 µl of Triton X-100 (10%, v/v in water) to the liposome suspension, which caused total disruption of vesicles. Leakage was calculated by the equation:
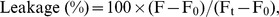
where F and F_t_ are fluorescence before and after addition of detergent and F_0_ the fluorescence of intact vesicles [38].

### Protease Sensitivity Assay

Tetrabranched M33-L or M33-D peptides (300 µg) were incubated at 37°C with *Staphylococcus aureus* aureolysin (3 µg, BioCol GmbH) or *Pseudomonas aeruginosa* elastase (3 µg, Calbiochem) in 300 µl 20 mM Tris-HCl, 1 mM CaCl_2_ pH 7.8. At indicated time intervals, 50 µl aliquots were removed, diluted with 950 µl of 0.1% trifluoroacetic acid (TFA)/water and analyzed by HPLC and mass spectrometry. Liquid chromatography was performed on Phenomenex Jupiter C18 analytical column (300 Å, 5 µm, 250×4.6 mm) in a 30 min gradient, using TFA 0.1%/water as solvent A and methanol as solvent B. Mass spectrometry analysis was performed on withdrawn samples and repeated on HPLC-eluted peaks with a Bruker Daltonic ultraflex MALDI TOF/TOF mass spectrometer.

### Anti-biofilm Activity

Biofilm formation was performed by adapting the procedure described in [39] using the Calgary Biofilm Device (Innovotech, Innovotech Inc. Edmonton, Canada). Briefly, 96-well plates containing the bacterial inoculum were sealed with lids bearing 96 pegs on which the biofilm could build up. The plates were placed in an orbital incubator at 35°C (for *P. aeruginosa* and *E. coli*) or 37°C (for *S. aureus*) for 20 h under agitation at 125 rpm. Once biofilms formed, the lids were removed from the plates and the pegs were rinsed twice with phosphate buffered saline (PBS) to remove planktonic cells. The peg-lid was then transferred to a 96-well challenge microtiter plate, each well containing 200 µl of a twofold serial dilution of each peptide in LB medium. The challenge plate was incubated at 37°C for 2 hours. Peptide activity on pre-formed biofilm was evaluated by two independent methods: (i) visual observation of bacterial growth and (ii) counting of living bacterial cells after peptide treatment. In the first case, the peg-lid was removed from the challenge plate, rinsed with PBS and used to cover a 96-well recovery microtiter plate, each well containing 200 µl LB medium. The recovery plate was sealed, incubated at 37°C for 4 hours and then observed for any visible growth of bacteria detached from the peptide-treated biofilm. Growth of bacteria in a particular well indicated regrowth of planktonic cells from surviving biofilm. Minimum biofilm eradication concentration (MBEC) was defined as the minimum peptide concentration preventing regrowth of bacteria from the treated biofilm within 4 hours.

In the second case, to determine viable cell counts of biofilms after peptide treatment, pegs from the challenge microtiter plate were removed and transferred to Eppendorf tubes containing 500 µl PBS. After sonication at room temperature for 15 min to break up the biofilm and remove bacterial cells from the peg, aliquots of bacterial suspension were plated on LB-agar plates for counting. Colony forming units (CFU) were expressed as percentage with respect to control (peptide-untreated biofilms). Minimum bactericidal concentration (MBCb) was defined as the lowest peptide concentration required to reduce the number of viable biofilm cells by ≥3 log_10_ (99.9% killing) [40].

### *In vivo* Experiments

Animal procedures were approved by the Ethical Committee of the Azienda Ospedaliera Universitaria Senese on November 18, 2010. Balb-c mice (20 g) were infected i.p. with lethal amounts of bacteria (see results) mixed in 500 µl PBS +7% mucin (mucin from porcine stomach, type II, Sigma-Aldrich). Bacteria were cultured overnight, centrifuged, mixed in sterile PBS, and measured by spectrophotometer. Possible further dilutions in PBS were sometimes necessary to obtain the right amount of bacteria. Groups consisted of 5 animals. Moribund animals were killed humanely to avoid unnecessary distress. Surviving mice were monitored for 7 days. Thirty minutes after bacterial administration, peptides were inoculated i.p. with 0.5 ml PBS solution containing the indicated amount of peptide (see Results). Control animals received only PBS. P values were calculated using GraphPad Prism software.
